# An Extra Breath of Fresh Air: Hyperbaric Oxygenation as a Stroke Therapeutic

**DOI:** 10.3390/biom10091279

**Published:** 2020-09-04

**Authors:** Blaise Cozene, Nadia Sadanandan, Bella Gonzales-Portillo, Madeline Saft, Justin Cho, You Jeong Park, Cesar V. Borlongan

**Affiliations:** Department of Neurosurgery and Brain Repair, University of South Florida Morsani College of Medicine, 12901 Bruce B Downs Blvd, Tampa, FL 33612, USA; bcozene@tulane.edu (B.C.); nas146@georgetown.edu (N.S.); bellagonzales-portillo2024@u.northwestern.edu (B.G.-P.); saftmad@umich.edu (M.S.); justincho@usf.edu (J.C.); youjeongpark@usf.edu (Y.J.P.)

**Keywords:** preconditioning, stem cells, cerebral ischemia, hyperbaric oxygen therapy, neuroprotection

## Abstract

Stroke serves as a life-threatening disease and continues to face many challenges in the development of safe and effective therapeutic options. The use of hyperbaric oxygen therapy (HBOT) demonstrates pre-clinical effectiveness for the treatment of acute ischemic stroke and reports reductions in oxidative stress, inflammation, and neural apoptosis. These pathophysiological benefits contribute to improved functional recovery. Current pre-clinical and clinical studies are testing the applications of HBOT for stroke neuroprotection, including its use as a preconditioning regimen. Mild oxidative stress may be able to prime the brain to tolerate full extensive oxidative stress that occurs during a stroke, and HBOT preconditioning has displayed efficacy in establishing such ischemic tolerance. In this review, evidence on the use of HBOT following an ischemic stroke is examined, and the potential for HBOT preconditioning as a neuroprotective strategy. Additionally, HBOT as a stem cell preconditioning is also discussed as a promising strategy, thus maximizing the use of HBOT for ischemic stroke.

## 1. Introduction

Strokes occurs primarily due to interruption of blood flow to the brain and is typed as ischemic or hemorrhagic. Ischemic strokes make up approximately 87% of total strokes [1]. Currently, the only FDA approved treatment for ischemic stroke is thrombolytic tissue plasminogen activator (tPA) for dissolving the blood clot and improving blood flow in the brain. When tPA is administered intravenously up to 4.5 h after stroke onset in patients with acute ischemic stroke, it reduces mortality and increases rates of independent ambulation when thrombolytic treatment is given early. If tPA is not delivered within the given time frame, it becomes associated with increased occurrences of severe hemorrhagic transformation, thereby limiting the therapeutic opportunity [2]. Since stroke is currently the fifth leading cause of death in the U.S., and someone dies of one approximately every 4 min, the discovery of new and effective stroke treatments is of high necessity moving forward.

An ischemic stroke is caused by abrupt blood vessel occlusion that causes ischemic damage to the area of the brain supplied by the occluded artery. During the acute phase of an ischemic stroke, excitotoxicity, oxidative stress, and microglial activation occurs, and causes neural death in the ischemic core [3]. In the penumbra, the apoptosis cascade is mainly triggered by the influx of calcium, which activates calpains and ultimately mitochondrial release of cytochrome c and eventually caspase-3 [4]. Furthermore, the intracellular calcium promotes mitochondrial dysfunction, generation of reactive oxygen species, and production of nitric oxide [5]. Also, an abnormal increase of water movement into the intracellular space occurs during the acute phase and results in edema [6]. In the subacute phase, aberrant neuroinflammation heightens the presence of matrix metalloproteases (MMPs), which cause an increase in blood-brain barrier (BBB) permeability, consequently allowing leukocytes to migrate to the damaged area of the brain and increase inflammatory activity [7]. The expression of reactive oxygen species-generating enzymes, like NADPH oxidases (NOXs), is also implicated in increased BBB permeability by promoting inflammation and dysfunction of endothelial tissue [5]. Other areas of the brain tissue surrounding the infarct area are vulnerable to the same exacerbating damage, but other cell phenotypes can be protected and serve as an area of focus towards monitoring the motor and cognitive functional deficits that precede a stroke [8].

Hyperbaric oxygen therapy (HBOT) serves as a potential non-invasive therapy where pure oxygen can be administered in a pressurized chamber at high levels of atmospheric pressure. With increased oxygenation, HBOT can be used to improve oxygen flow from lungs to systemic organs and can reduce secondary brain injury effects, including apoptotic pathway initiation, oxidative stress, and rampant inflammation [9]. By restoring oxygen tension, HBOT has been shown to restore cellular energy production, stabilize cellular calcium, decrease NOXs expression, and attenuate oxidative stress [10]. Mechanisms reached with hyperbaric oxygen can also be achieved at lower or normal oxygen concentrations [11]. During the recovery stages, where oxygen levels are close to normal, HIF-1 alpha synthesis increased, and a relatively hypoxic environment existed [11]. In this setting, MMP, a hypoxic stimulus [12], and EPO production elevated as well, which was also observed in healthy human subjects. This suggests that similar mechanisms of HBOT can be demonstrated with lower tensions of oxygen. Additionally, HBOT has also proven beneficial in the treatment of other pathological diseases, including traumatic brain injury (TBI), spinal cord injury (SCI), and stroke [13]. Currently, there are few treatment options available for many neurological diseases; however, experimental studies utilizing HBOT demonstrate promising results. HBOT studies target ameliorating ischemic-related damage to improve the quality of life of affected patients. In this review, the use of HBOT for ischemic stroke will be covered in-depth, including information on the application of HBOT preconditioning in the stroke brain and the potential of HBOT priming for stem cell transplantation. Here, we provide the pre-clinical bases for advancing the use of HBOT as a promising target for stroke therapy.

## 2. HBOT in Ischemic Stroke: Potential Benefits and Limitations

In early investigations on hyperoxia following a stroke, HBOT was seen as a dangerous apparatus with few beneficial properties and was deemed to have no therapeutic value. Nevertheless, in the last 20 years, expansive research has demonstrated the ability of HBOT to reduce the severity of infarct volume and serve as a potential treatment option [14]. However, HBOT must be administered during the small reperfusion window, which creates a limited opportunity for effective treatment. This has been a reoccurring problem with HBOT because of the difficulty associated with determining the reperfusion window and extensive imaging to determine the appropriate course of action [15]. Evidently, some studies indicate that delayed HBOT past the effective window of treatment actually causes worse outcomes. This is suggested to be due to the role of ROS in glutamate-induced excitotoxic cell death [16]. Therefore, further research on the use of HBOT must be conducted and carried out safely to prevent treatment with excess oxygen that could cause additional harm, including obstructive pulmonary disease [16].

Acute ischemic stroke (AIS) serves as a primary cause of death worldwide and is characterized by an occlusion of the cerebral artery. The therapeutic treatment plans for AIS are, however, limited to date. HBOT treatment serves as an option to improve AIS-induced brain tissue hypoxia [17]. The primary goal of acute HBOT treatment is to increase oxygen levels in the ischemic region during stroke occlusion, in pursuit of minimizing hypoxic damage. Studies have demonstrated the ability of HBOT to be used to enhance the arterial partial pressure of oxygen [18], increase the oxygen content [19], stabilize the blood-brain barrier [20], decrease the intracranial pressure, and relieve cerebral edema. Moreover, HBOT serves as a safe practice for the treatment of acute ischemic stroke moving forward.

In regard to chronic ischemic stroke, HBOT demonstrates limited potential in treating chronic neurological deficits. Nonetheless, in a recent study, the efficacy of HBOT in restoring memory function for chronic stroke patients revealed significant memory improvement and increased brain metabolic rate [21]. Additionally, patients experienced significant improvement in memory and attention testing scores [22]. Studies have demonstrated that the earlier HBOT is initiated, the greater its therapeutic effectiveness [23]. Clinical trials have demonstrated consistent safety, but inconsistent efficacy. Limited studies have demonstrated the efficacy of HBOT for the treatment of chronic ischemic stroke, and the data is still unreliable for further use in patients.

Another potential treatment for stroke that has been explored is the use of normobaric oxygen, which its therapeutic efficacy has been both reported and refuted [16,24,25]. The normobaric oxygen paradox (NOP) was introduced to describe a potent mechanism where Hypoxia Inducible Factor 1 alpha (HIF-1 alpha) expression is regulated by oxygen [26]. Because HIF-1 alpha is responsible for erythropoietin (EPO) and VEGF expression, normobaric oxygen may be correlated to EPO production [27]. Studies hypothesize that sudden return to normoxia after a small exposure to normobaric hyperoxia would result in a relative hypoxia state [27,28]. This, in turn, would elevate EPO production because hypoxia is a natural trigger for EPO production [26,28]. Additionally, relative hypoxia induced by oxygen gradient and NBO may induce reticulocyte production [28]. Normobaric oxygen therapy increases dissolved oxygen levels within the bloodstream, but displays controversial neuroprotective effects. Specifically, critical information regarding the optimal therapeutic time frame of NBO and its long-term effects are still unclear Therefore, it does not serve the same effectiveness as the HBOT for post- and pre-stroke conditions and is less of an intriguing option for preconditioning [16,24,25]. Nevertheless, the synergistic use of normobaric oxygen treatment accompanied by HBOT shows promise in reducing stroke mortality [2,29]. Recent in vivo investigations have shown that HBOT and normobaric oxygen treatment may widen the limited window for thrombolytic therapy, and various neuroprotective medications [29,30].

Currently, HBOT has been used as a post-stroke therapy as well as a preconditioning method. The goal of HBOT is to stimulate a hyperoxia environment during ischemic and reperfusion periods following a stroke. Additionally, HBOT may expose a patient to recurrent treatments after the initial early treatment time-frame has passed [31]. Preconditioning treatments have focused on exposing an individual to a mild stressor, which is supposed to enhance the brain’s resistance to future stressors [32].

### 2.1. Preclinical Functional Outcomes of Post-stroke HBOT

Typically, HBOT treatment is carried out at 2.5 atmospheres (ATA) for 60–90 min [33]. The atmospheric pressure must not exceed the given 2.5 ATA during HBOT, or it could cause oxygen toxicity, increase oxidative stress throughout the body, or increase the risk of seizure activity [31]. The goal of HBOT treatment is to increase the oxygen concentration in the ischemic region of the brain to minimize hypoxic damage. If administered after the initial treatment window, continuous HBOT treatments could promote the stimulation of endogenous repair processes [34].

HBOT is highly effective when administered 30 to 60 min after stroke, and the potency of treatment lessens if initiation is delayed any further. HBOT exhibits therapeutic potential in its ability to reduce infarct volume and improve behavioral scores in patients [14,15]. Research has demonstrated different time points at which HBOT is effective, such as HBOT (2.5 ATA for 2 h) at 6 h after reperfusion [35], and HBOT (3 ATA, 1 h) at 3 and 6 h after reperfusion [36]. Additionally, single-session HBOT is reported to be effective up to 18 h and 48 h after stroke [37]. In order to determine the therapeutic effects with HBOT at various timepoints, extensive and tailored treatment protocols are required. On the other hand, delayed treatment with HBOT (2.5 ATA, 2 h) at 6 or 24 h after stroke results in an increase of infarct size and causes neurological deficits [38]. Little evidence has proven the effectiveness of HBOT that is given more than 48 h post-stroke. One study has used one session of HBOT (3 ATA, 1 h) and exhibited significant neuroprotective effects when delivered 72 h post-stroke [20]. Therefore, it is imperative to deliver HBOT within the given time-frame to ensure the best outcomes.

### 2.2. Clinical Results of HBOT in Stroke

Many clinical trials have explored the use of HBOT, but have revealed inconclusive results [39]. Various factors, including the unstable clinical status of the acute stroke patients, could be the cause for varying results and prevent the patients from the given effective treatment window that is required 3–5 h after reperfusion [40]. Additionally, many treatment protocols could be different and take a wide variety of patients that could hinder the ability to draw reliable conclusions and comparisons between groups. It is imperative to acknowledge the fact that clinical success for HBOT has been achieved, and the use of HBOT has been shown to lessen levels of cerebral and myocardial biomarkers and reduce the length of stay in an intensive care unit [41]. Moreover, the clinical significance of HBOT should not be overlooked, and further research should be carried out [42]. A growing number of clinical trials have been performed and demonstrate cerebral plasticity, as well as the ability of HBOT to restore memory function and improvement following a chronic stroke [21]. The timing of the HBOT application is critical. Conclusive studies have found that if HBOT is initiated earlier, it leads to greater therapeutic effectiveness [15,23]. After 12 h post-ischemia, the benefits of single treatment HBOT are reduced. However, repetitive applications of HBOT in the sub-acute stroke brain document neurogenic effects [43]. Studies have even demonstrated a consistent safety and the potential of using HBOT for chronic stroke patients, a breakthrough finding that was thought of only as detrimental previously. Nevertheless, contradicting results in the use of HBOT for acute and chronic stroke patients warrant further exploration to clear up the inconsistencies in studies.

## 3. Unpacking Mechanisms of Action of HBOT in Stroke

### 3.1. Physiological and Metabolic Effects

The goal of HBOT is to increase perfusion and oxygenation of at-risk tissue. HBOT therapy can be used to enhance arterial oxygen saturation, and augment tissue oxygen content via enhanced cerebral microcirculation [18,31]. Additionally, HBOT serves a vital role in enhancing BBB stability through MMP regulation [23], and can also decrease intracranial pressure and relieve cerebral edema [31]. Secondary effects of HBOT in the ischemic brain may be prevalent, due to the reduction of extracellular glutamate levels, causing neural dysfunction and excitotoxic death [44].

### 3.2. Antioxidant Effects

HBOT demonstrates the ability to provide oxidative protection against stroke-induced ROS and nitrosative species [45]. This startling discovery reverts the fact that introducing high levels of oxygen can actually induce oxidative stress and exhibits the efficacy of HBOT. Following a stroke, HBOT has been proven to reduce levels of pro-oxidative enzymes, including malondialdehyde and increase the activity of CAT and SOD [45]. Additionally, other studies have found reduced stroke-generated ROS in the striatum after HBOT therapy [46]. HBOT also plays an effect on nitric oxide synthase and provides antioxidant protective properties [47]. However, various experimental designs and different durations of treatment sessions may hinder a complete understanding of the role of the HBOT for reducing oxidative damage. It is important to note that HBOT treatment may cause oxidative stress, but can be balanced by antioxidant mechanisms. Further studies are required to determine the effects of HBOT on oxidative pathways.

### 3.3. Anti-Inflammatory Effects

Aberrant inflammation is a key player in the pathogenesis of stroke, and catalyst for secondary cell-death in the brain. Interestingly, HBOT has been linked to anti-inflammatory effects in the setting of ischemic stroke. Experimental stroke studies have shown decreases in markers of inflammation, such as tumor necrosis factor-alpha and CD40+ microglia in HBOT-treated animals [48,49]. Although the mechanism underlying the anti-inflammatory effects of HBOT has not been fully elucidated, pre-clinical studies showed reduced secretion of inflammatory chemokines, which inhibit the accumulation of leukocytes at the ischemic area [17]. Similarly, another study showed HBOT reduced myeloperoxidase activity and inhibited neutrophil infiltration of the injured tissue [50].

### 3.4. Additional Neuroprotective Mechanisms

HBOT has been linked to many pathways that preserve neural tissue and reduce apoptosis as displayed in Table 1. Mechanisms mentioned previously in this section many be heavily related to these as neuronal apoptosis is prompted via metabolic restriction, inflammation, and ROS. Undiscovered pathways may also contribute to HBOTs efficacy. Notable findings include a lower concentration of cortical and hippocampal caspase-3 [51], reduced HIF-1α [52], augmented growth factor levels of GDNF and NGF [53], and regulation of mitochondria [54]. Furthermore, HBOT has been seen to directly modulate glial cells, consequently contributing to the protection of vulnerable neurons [55].

## 4. Implications of HBOT in Other Neurological and Non-Neurological Conditions

### 4.1. HBOT in Acute and Chronic TBI

HBOT has been shown to be a safe and effective treatment for TBI, specifically when conducted during the acute phase [53,56,57,58]. Utilizing a rodent model of TBI, HBOT initiated at 3 h after cortical deformation induction attenuated symptoms after histopathological analysis [59]. Another murine model of TBI offered evidence that HBOT-induced cognitive and learning regenerative abilities were preserved from 3 h after injury to 7 days [13]. While experimental protocols, such as therapeutic windows, may vary, HBOT consistently provides neuroprotection for acute TBI [60]. Nonetheless, HBOT may also translate to functional benefits in chronic TBI as well. A human TBI-HBOT study indicated increased cerebral perfusion and angiogenesis and ameliorated memory ability, executive function, information processing speed, and global cognitive scores up to 27 years’ post-injury [61]. Although many studies have found similar therapeutic effects of HBOT in TBI [62,63,64,65], other studies have not supported these findings [66].

### 4.2. HBOT in Spinal Cord Injury

Overwhelming evidence has indicated that cell death responses induced by secondary injury contribute more significantly to SCIs than primary trauma [67]. The following mechanisms are associated with secondary cell damage and can potentially be ameliorated by HBOT: Reactive oxidative injury, astrocytic glial scarring, glial penetration, and lymphocyte/activated monocyte/phagocytic macrophage generation [68]. Importantly, HBOT engenders neuroprotection in SCI via attenuation of anoxia and heightened rehabilitation of neurons [69]. HBOT correlates with decreased oxidative enzymes, apoptotic factors, and inflammatory agents [70]. In addition, HBOT has been shown to safeguard BBB function and improve motor dysfunction post-SCI [71]. Notably, SCI patients who underwent HBOT, demonstrated substantial amelioration of neurological injury and maintained neuronal functions, such as induced potential amplitude and conduction velocity, more effectively than the control group [72]. The mechanisms behind HBOT-induced neuroprotection may involve the upregulation of vascular endothelial growth factor (VEGF) expression, restoration of axons, and inhibition of apoptosis [73].

### 4.3. HBOT in Other Pathological Contexts

Several studies have elucidated that HBOT bears curative potential in a multitude of disorders that manifest in oxidative stress, inflammation, and ischemic injury [74,75,76,77]. HBOT has been explored as a therapeutic regimen in patients suffering from diseases that entail hypoxia of tissues, such as diabetic ulcers [78,79] and acute coronary syndrome [80,81]. Evidently, the therapeutic abilities of HBOT may encompass tissue repair processes specific to the disease. For instance, HBOT imparts antimicrobial capacity in necrotizing tissue contamination [82] and enhances gas dissolution in air embolisms [83]. In addition, HBOT has shown promising results in psychological pathologies, such as post-traumatic stress disorder (PTSD) and post-concussive malady, that may arise following brain damage [63]. After 40 HBOT treatments (15 ATA, 60 min) conducted within 30 days, PTSD manifestations were significantly alleviated [62,84]. HBOT therapy ameliorated cognitive deficits, diminished anxiety and depression, and augmented blood circulation via the cerebrovasculature into the white matter [65]. Additionally, spontaneous physiological irregularities like autism spectrum illnesses may be potential therapeutic targets for HBOT [85]; however, HBOT’s neuroprotective effects in these disorders warrant further investigation [86]. In light of the current COVID-19 pandemic, clinical trials have explored HBOT in infected patients experiencing extreme respiratory distress. COVID-19 infection generates a potent cytokine storm that spurs the onset of progressive hypoxia, leading to severe respiratory illness [2,87]. Furthermore, HBOT’s capacity to enhance oxygen flow and attenuate inflammation may bear curative effects in COVID-19 patients [87]. In Wuhan, HBOT was administered to critically ill COVID-19 patients and resulted in ameliorated hypoxia, increased blood flow, and mitigated immune system deficits. Remarkably, these patients demonstrated substantial recovery and were released from hospital care after 3–8 HBOT sessions [2]. In another clinical trial, COVID-19 patients demonstrated alleviated shortness of breath [87], though a significant reduction in mortality rate was not observed. Therefore, the potential of HBOT in COVID-19 treatment warrants further clinical investigation. As copious evidence suggests, HBOT similarly affords a stable safety profile in non-stroke diseases when it is administered properly.

## 5. Pre-Clinical Findings with HBOT Preconditioning for Stroke

HBOT preconditioning has been shown to provide therapeutic benefit in neurological diseases like ischemic stroke [42,88]. The mechanism behind the protection is attributed to the introduction of mild oxidative stress, which builds tolerance in endogenous cells to future insult. Patient populations with a higher risk for ischemic strokes, such as those with comorbidities (e.g., obesity, diabetes, hypertension, atherosclerosis, etc.), may benefit from preemptive HBOT, and advancements in imaging techniques that allow for more accurate prediction of stroke risk may increase the value of preconditioning therapies [89].

The first pre-clinical study evaluating the effects of HBOT preconditioning on a gerbil ischemic stroke model showed that the therapy conferred tolerance to ischemia and prevented neuronal death [90]. The following studies using other animal models showed that HBOT was protective against transient, not permanent, stroke, and protection was conferred in a dose-dependent manner [91]. A treatment of five sessions (2.5 atmosphere absolute [ATA], 1 h) over consecutive days was more effective than three sessions at rescuing functional deficits in rats after middle cerebral artery occlusion (MCAO) 24 h after the last session [91]. These results have been replicated by other studies, including one that showed that four sessions of HBOT (2.5 ATA, 1 h, twice a day) offered neurological and histopathological protection from MCAO 24 h after the last session [92]. More aggressive treatments (3.5 ATA, 1 h, five consecutive days) have also provided significant histopathological signs of neuroprotection [93]. Other pre-clinical studies have explored the therapeutic window for HBOT, which suggests neuroprotection can be achieved by treatment 24 h before ischemia, but not 72 h [94]. However, it is important to note that intensity and number of sessions may play a bigger role in treatment effects, and the therapeutic window must be further investigated. In the following sections, we will discuss the potential mechanisms underlying neuroprotection provided by HBOT preconditioning.

### 5.1. Preparation for Oxidative Stress

HBOT primarily generates neuroprotection through its interactions with an oxidative preconditioning factor [95]. Long-term subjection to hypoxic environments engenders drastic oxidative strain, the disarray of the antioxidant network, and ultimate cell damage. Initial exposure of brain tissue to non-lethal hypoxia via HBOT preconditioning can shield neurons from future ischemic injury by fortifying tissue against oxidative stress. As evidence suggests, oxidative toxicity may be spurred by ROS and reactive nitrogen species (RNS) (e.g., peroxynitrite and NO_2_) upregulation in the CNS. Fortunately, cells can combat ROS escalation through defense mechanisms induced by antioxidant enzymes. Superoxide dismutase (SOD) sequesters superoxide, catalase/peroxidases neutralize hydrogen peroxide, and glutathione S-transferase off set lipid peroxides. Auxiliary enzymes, such as glutathione reductase (GRX) and glucose-6-phosphate dehydrogenase (G6PD), also contribute to brain tissue defense. Although sparse numbers of ROS can be beneficial, as they promote antioxidant enzyme pathways that make up the adaptive cellular response, lethal amounts of ROS, engendered by hyperoxia, surpass the cellular antioxidative potential and generate oxidative damage. Ischemic injury in the brain induces oxidation of proteins, lipid peroxidation and augmented DNA mutation, leading to cell membrane impairment, disturbances in metabolism, and tissue death [96]. ROS levels are higher with HBOT, due to increased oxygen partial pressure and upregulated H_2_O_2_ produced by mitochondria [42]. Moreover, non-lethal oxidative stress spurred by HBOT serves as a protective procedure, stimulating antioxidative activity [95].

An in vivo investigation utilizing a focal cerebral ischemia model found that HBOT preconditioning elevated SOD and CAT mechanisms in cerebral tissue, leading to increased survival rates, as well as ameliorated neurological function and cell damage [92]. Notably, the stroke-afflicted penumbra and hippocampus demonstrated diminished levels of lipid peroxidation and oxidative stress biomarker, malondialdehyde (MDA) [92]. In the same way, HBOT preconditioning with a spinal cord ischemia experimental model upregulated SOD and CAT processes. However, activation of the CAT inhibitor, 3-amino-1, 2, 4- triazole, prior to ischemic stroke, abolished the beneficial effects of HBOT, like spinal cord resilience to oxidative stress decreased significantly [97]. When dimethylthiourea, a free radical scavenger, was delivered to the spinal cord ahead of HBOT, the elevated SOD and CAT activity was eliminated [97]. Moreover, HBOT preconditioning spurs preliminary oxidative stress that prompts antioxidative mechanisms from enzymes, leading to increased resistance of tissue to future ischemic damage.

By suppressing GRX and G6PD and elevating glutathione peroxidase (GSH-Px) and glutathione S-transferase (GST) pathways, frequent non-lethal HBOT preconditioning imparts neuroprotection against oxidative damage in the central nervous system [98]. Therefore, in an indirect manner, HBOT attenuates oxygen toxicity through the repression of G6PD mechanisms. Importantly, HBOT preconditioning bolsters antioxidative processes and dilutes enzymatic activity of pro-oxidants, as HBOT-induced diminishment of G6PD can be correlated with the truncation of GRX and the augmentation of GSH-Px activity.

Under normal conditions, HBOT promotes neuronal rehabilitation and neuroprotection by upregulating heat shock proteins (HSPs), particularly HSP70 [99]. HSP70 inhibits protein build-up, restores slightly denatured proteins, attenuates inflammation, and hinders apoptosis, all of which contribute to neuroprotection [100]. In addition, as displayed in vitro, HBOT fortifies the expression of HSP32, shielding neuronal tissue from oxidative damage, and oxygen-glucose deprivation (OGD) [92,101]. HSP32 or heme oxygenase-1 breaks down heme into carbon monoxide (CO), biliverdin, and ferrous iron. Hemoprotein oxidation, such as hemoglobin, myoglobin, and neuroglobin oxidation, engender the formulation of free heme. An iron atom lies in the middle of the heme molecule and can interact with H_2_O_2_ to form deleterious hydroxyl radicals. HSP32 catalyzes heme molecules, leading to the generation and build-up of ferritin release, which in turn, engenders the removal of iron, thereby safeguarding tissue from oxidative injury [92,101]. Importantly, HSP32 is known to be incited by ROS and nitric oxide (NO), as the ROS/p38/MAPK/Nrf2 pathway and MEK1/2Bach1- regulated negative feedback modulate HSP32 activity [102]. Moreover, free radical production spurred by oxidative stress should promote HSP mechanisms. At very low concentrations, free radicals produced by mitochondria and NOXs serve a regulatory ole in cellular activity—their physiological role in cellular activity ties in with the use of HBOT. Nonetheless, evidence points to the idea that response to HBOT may be cell-specific. For instance, one HBOT subjection in healthy males demonstrated no elevation in HSP72 activity in peripheral blood mononuclear cells (PBMC) [103].

Additionally, HBOT may impart neuroprotection against oxygen toxicity via an increase in NrF2-mediated antioxidant gene expression. Notably, more than 200 antioxidants and cytoprotective genes can be turned on by the Nrf2 pathway [104]. HBOT not only promotes Nrf2 activity, but also upregulates essential proteins involved with intracellular GSH production and transport (e.g., GST, MRPI, GCL, cGT), the assembly/disassembly of macromolecules (e.g., HSPA1A, HSP32), and antioxidant enzymes (e.g., SOD1, GST), which are all target genes of Nrf2 [31,102,105,106,107]. In addition, by bolstering the expression of SirT1 in more than three various ways, HBOT preconditioning imparts neuroprotection. Firstly, Sirt1 expression can be increased by mediating the fasting-induced initiation of Nrf2 signaling upstream through the regulation of the PPAR-ƴ/PGC1-1α complex that attaches to the Nrf2 promoter, stimulating expression. Secondly, SirT1 expression is mediated via repression of apoptosis, spurred by the upregulation of protein anti-apoptotic Bcl-2 expression, depletion of cleaved caspase-3, which is pro-apoptotic, and the removal of acetyl groups from p53. Thirdly, expression of SirT1 can be modulated through the augmentation of FoxO, which in turn, elevates SOD and CAT activity under oxidative stress [107,108,109,110].

**Figure 1:**
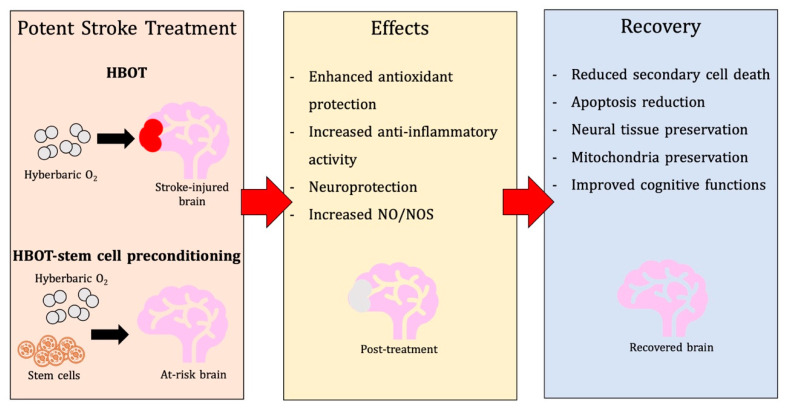
Hyperbaric oxygen therapy (HBOT) effects on Antioxidants and NO.

Indeed, HBOT preconditioning can be linked to the inflation of nitric oxide, [111,112] as shown in Figure 1. Serving as a key neurotransmitter, NO is generated by NO synthase (NOS) and is a critical agent of neuroprotection and neurotoxicity [113]. Following cerebral ischemia, endothelial NOS (eNOS) secretion of NO is beneficial, as it stimulates vasodilation. Conversely, once ischemia evolves further, NO generated by hyperactivity of neuronal NOS (nNOS) and iNOS expression lead to cerebral injury. NO released by eNOS and iNOS promote synaptic plasticity and neuronal development, whereas NO secreted by nNOS has the opposite effect, attenuating neurogenesis [114]. Since NO improves the vasodilation of the cerebrovasculature, it may fortify the oxygenation of tissues. Furthermore, NO possesses the capacity to favor or impair apoptosis. NO may also regulate cellular metabolism in the presence of dysfunctional mitochondria. On the other hand, it may escalate the transit of ROS to tissues as well. Additionally, NO may react with free radicals to generate toxic oxidant peroxynitrite and engender nitrosative injury. Importantly, preconditioning with HBOT elevates antioxidant enzymatic activity and represses peroxynitrite primarily in the hippocampus, demonstrating HBOT’s protective capabilities [98].

As low amounts of NO displays beneficial effects after stroke, high concentrations of NO produced via iNOS or eNOS may augment neuroinflammation and neurotoxicity. NO provides these negative effects through various mechanisms, including cGMP, cAMP, G-protein, JAK/STAT, and MAPK dependent pathways. Moreover, NO is also believed to modulate specific gene expression, further exacerbating inflammation, and toxicity [115].

Aside from NO’s capacity to cause inflammation, HBOT-induced upregulation of eNOS and nNOS mRNA and protein, along with increased NO in the hypothalamus and hippocampus, may amplify convulsion susceptibility following consecutive HBOT subjections, which may exacerbate the risk of seizures in successive HBOT exposures [112]. Notably, the nonspecific NOS inhibitor, L-NAME, eliminated HBOT-induced neuroprotection, indicating that elevations in Mn-SOD, CAT, and Bcl-2, as well as apoptosis inhibition, may be regulated by NO [69]. Furthermore, following HBOT preconditioning, NO bears both neuroprotective and neurotoxic effects, and thus, further investigation into the mechanisms of NO after HBOT pre-treatment is warranted.

### 5.2. Reduction of Apoptosis, Activation of Autophagy, and Promotion of Cell Survival

ROS molecules possess the ability to react with molecular components to initiate apoptosis or necrosis. Inhibiting major redox systems, such as thioredoxin reductases (TrxR), results in the production of ROS and increased cell apoptosis [116]. PTSD models in rats revealed the upregulation of TrxR-1 and TrxR-2 mRNA in the hippocampus in addition to decrease levels of apoptosis of neurons after HBOT [117]. Additionally, HBOT preconditioning reduced cellular necrosis by modulating mitochondrial pathways. Specifically, cytoplasm cytochrome c levels, as well as capase-3 and capase-9 activity were reduced, upregulating Bcl-2 and Bax proteins linked with improved brain recovery [93,118,119,120,121]. Inducing BDNF and inhibiting p38/MAPK phosphorylation also reduced the early onset of apoptosis and apoptosis progression [93,122]. Therefore, HBOT preconditioning in stroke evidently limits apoptosis progression by promoting anti-apoptotic activity and protein expression.

In addition to initiating apoptosis, ROS also moderates starvation-induced autophagy via class III phosphoinositide 3-kinase pathway, which sabotages the survival mechanism. ROS-induced autophagy was demonstrated when HBOT preconditioning upregulated protein expression levels of LC3-II and Beclin 1, causing autophagosomes to form in the ischemic penumbra post-ischemia in rat brain models [123]. Additionally, HBOT preconditioning enhanced cell survival by downregulating MMP-9 expression, inhibited CA1 cell damage, and promoted healthy functional performance [122]. Furthermore, preconditioning HBOT can activate Wnt signaling pathway, upregulate HIF-1, and secrete vascular endothelial growth factor (VEGF) to mitigate cell loss. HBOT increased levels of VEGF, VEGFR2, MEK1/2, Raf-1, and phosphor-extracellular signal-regulated kinase (ERK) ½ protein that further improved neurological functions [123].

### 5.3. Immunosuppression and Immunopreparation

Interestingly, HBOT preconditioning has been observed to reduce and even prevent aberrant inflammation by lowering neurotoxicity microglia activity, TNF-α expression, and neuronal degeneration [42], resulting in improvement in motor function after intracerebral hemorrhage [123]. Additional mechanisms include downregulation of expressions associated with post-ischemic neuroinflammation, such as cyclooxygenase-2 (COX-2), and alleviates cognitive impairments and physiological dysfunctions by restricting pro-inflammatory cytokines and caspase-3 activity in the hippocampus [55,124]. Based on these findings, HBOT preconditioning also alleviates cognitive impairment and protects brain functions by modulating pro-inflammatory cytokines and caspase-3 pathways [55,124].

### 5.4. Preservation of Blood-Brain Barrier, Edema Minimization, and Angiogenesis

HBOT demonstrated preservation of the blood-brain barrier (BBB) and minimizes edema after the onset of surgical brain injuries (SBI), stroke (either ischemic or hemorrhagic), and TBI [42,125,126,127,128,129]. These protective mechanisms exist due to the suppression of inflammatory responses by lowering hemorrhage volumes and reducing NLRP3 inflammasome expression to recover cognitive functions. Furthermore, HBOT preconditioning also relieved neurological dysfunctions and reduced blood volumes by reducing HIF-1α, MMP-2, and MMP-9 [42,129]. However, HBOT preconditioning may overexpress HSP-70 in the hippocampus, which could lead to cognitive deficits and oxidative stress [130]. Preconditioning of HBOT could bring protective effects of microvascular endothelial cell protection by increasing Nrf2 and HSP32 activity. Recent studies, however, reveal the therapeutic effects of HBOT on infarction volume, BBB, and transformed hemorrhage in the absence of the mentioned proteins in the focal cerebral ischemia model [129].

HBOT preconditioning may reduce edema via downregulation of aquaporin 4 (AQP-4) expression, which possess the mechanism to hinder hemorrhage and preserve neural tissue [131]. Cultured astrocytes post-HBOT revealed an increase in AQP-4 and VEGF, demonstrating the ability to modulate BBB openings. This mechanism may introduce a possible treatment option for drug transportation into the CNS [132]. Additionally, HBOT promotes the p44/42 pathway to help prevents the development of brain edema post-intracerebral hemorrhage; the activation of the pathway correlates to the cerebral ischemic tolerance that was observed [125]. In vitro models highlight the protective abilities of HBOT for BBB integrity when occluding and ZO-1 activities were regulated in hypoxic settings [133]. Alongside protecting BBB integrity and minimizing edema, HBOT may also protect energy metabolism and tissue perfusion by stabilizing glucose levels, preventing glutamate levels from increasing, lowering lactate/pyruvate ratios, and increasing Ang-2 activity. Protecting energy metabolism gave therapeutic effects, including increased microvessel density, reduced brain injury, and alleviated post-ischemic neurological deficits [44,134].

### 5.5. Considerations for HBOT Preconditioning Protocols

Hyperbaricity of 2.5 ATA and 21% O2 is not enough to promote ischemic tolerance. Therefore, understanding the components of HBOT in both hyperoxia and hyperbaricity scenarios is crucial to induce tolerance against ischemic injuries [135]. Both preconditioning of HBOT and hypoxia possess similar efficacies in the neonatal brain. However, the unique defense mechanisms are used during oxidative stress [136]. Regular HBOT preconditioning consists of 2–3 ATA with 60–90 min of exposure with 24 h intervals [137,138,139]. Effective ischemic tolerance can be developed when dosed with HBOT for 3–5 days [91,135]. Although, HBOT also induced neuroprotection against brain injuries, due to ischemia during a certain time frame [94], neuroprotection was achieved through biphasic time frames characterized by instant and delayed preconditioning effect [42,140]. Instantaneous preconditioning was evident within the first hour after treatment, demonstrating alterations in ion channel activities, enzyme activity, and secondary messengers [140]. On the other hand, delayed preconditioning involved cellular changes that progressed slower and developed enduring changes in gene and protein expression [140].

## 6. HBOT-Primed Stem Cells as a Promising Therapy

Stem cells located within niches of the matured brain possess protective and restorative capabilities for the brain post-stroke via migration to damaged sites. Stem cells are armed with mechanisms, such as secretion of angiogenic factors, trophic factors, regulation of cell death pathway, anti-inflammatory molecules, and replacement of damaged neuronal tissues [3]. However, limitations exist that prevent stem cells from engaging unaided stroke recovery, making therapeutic strategies that promote the brain’s reparative capabilities appealing. Enhancement of HBOT effects on stem cells is becoming more evident in post-stroke recovery studies [141], proposing a potent role of HBOT in stem cell conditioning before cell transplantation.

### 6.1. HBOT Effects on Endogenous Stem Cells

In vivo studies reveal the profound effects of HBOT on stem cell populations as shown in Table 2. Most prominent examples include increased amounts of endogenous stem cells via enhancement of stem cells in a pressure-sensitive environment [141,142] and multiplication of neural stem cells in adult brain niches [143], which have been observed in various models of TBI-induced injuries [144] and non-oxygen-induced injuries [145]. Alongside neural stem cell proliferation, HBOT possesses the ability to target damaged sites [123], proposing HBOT therapeutic effects that are not dependent on disease pathologies. During circulation, vasogenic endothelial progenitor cells (EPCs) and other specific stem cells released post-HBOT may demonstrate therapeutic effects on the stroke-injured brain [146]. Stem cells located locally or peripherally in the brain may induce treatment benefits when combined with mechanisms of HBOT preconditioning and HBOT mechanisms in stroke.

An accumulation of studies has demonstrated growing interest in the mechanisms of HBOT that enhance bone marrow-derived stem cell circulation [145]. Investigations experimented with nitric oxide synthase inhibitors and mice administered with eNOS [141], which revealed the elusive nature of HBOT mechanisms in cerebral neurogenesis upregulation. Among the various signaling and growth molecules, HIF-1 alpha was stabilized by HBOT, inhibiting activation of hypoxia [147], slowing down prolyl hydroxylase-induced degradation [148], and enhanced signaling of Wnt/beta-catenin pathways that raised levels of active neural stem cells [149]. High levels of ROS during NSCs proliferation may also induce their own renewal, suggesting drug inhibition of ROS may downregulate ROS reproductive activity [150] and enhancement of pro-NSC signaling pathways to promote NSC survival.

Vascular endothelial growth factor (VEGF), another highly important regulatory molecule, and its receptors, VEGFR2, ERK, and CREB, may be involved in HBOT-induced NSC proliferation, due to their significance in neurogenic pathways [151]. Targeting VEGF downregulates HIF-1 alpha, which is responsible for promoting hypoxia- and ischemia-related genes and expressions via inflammation, proliferation, glycolysis, and angiogenesis [42,148]. Past studies have demonstrated nontherapeutic effects of upregulating erythropoietin (EPO), another HIF-1 alpha gene target, in the hippocampus and cerebral cortex, including BBB permeability prevention, brain edema reduction, decreased infarction volume, and post-HBOT neurobehavioral improvement [152,153]. On the other hand, reduced EPO levels via HBOT promoted homing and engraftment mechanisms of transplantation of stem cells derived from the umbilical cord blood [154]. Furthermore, an increase in HIF-1 alpha expression was correlated to CXCR4 upregulation after HBOT [113], promoting neural crest stem/progenitor cells (NCSCs) via inhibition of hypoxia-involved signaling receptors. Cytoplasmic activity of TPM1 increased, and TP53 and CDKN1A, a cyclin-dependent kinase inhibitor, decreased following HBOT, which led to lower rates apoptosis and higher rates of NCSCs reproduction [113].

### 6.2. HBOT and Exogenous Stem Cells

The combination of HBOT and stem cell transplantation may reveal underlying mechanisms among these treatments. This concept has been researched in neurological and non-neurological settings, including TBI [155], SCI [156], and diabetes mellitus [157]. HBOT has been found to promote graft survival in the bone marrow, peripheral blood, and spleen. These results were discovered after an umbilical cord blood stem cell transplantation present in a rodent model of whole-body irradiation injury [158]. In a murine SCI model, enhanced MSC graft survival was revealed in animals with combined HBOT and cell therapy [156]. These animals also displayed an alleviated inflammatory response, including decreased levels of pro-inflammatory mediators, TNF-α, IL-6, and IFN-α [156]. Results from another study show a suppressed inflammatory response along with an upregulated nerve regeneration and a decrease in expression of TUNEL, an apoptosis marker, in combination therapy animals [159].

### 6.3. Effects of HBOT In Vitro: Potential for Stem Cell Priming

Molecular signaling pathways, including Wnt/β-catenin, VEGF/VEGFR2, and CREB, are potentially mediated by non-stem cell host tissue secretions. While information gained from in vivo studies guide in vitro research, both stem cell and non-stem cell-mediated mechanisms must be analyzed. The negative effects of HBOT on stem cells must also be considered. HBOT reduced cell survival in mesenchymal stem cell (MSC) cultures [160]. The augmented oxygen tension expressed by HBOT subsequently enhances the formation of ROS and releases oxidative stress on cells [161,162].

Careful evaluation of HBOT priming stem cells for transplantation must be conducted, since exposure to oxidative stress in vitro may advance stem cell resiliency to oxidative stress after transplantation. In vitro HBOT priming potentially has genetic, molecular, or transcriptomic effects on stem cells, which enhanced their therapeutic viability. HBOT-induced oxidative stress may augment the resiliency of stem cells to the harmful post-ischemic brain environment.

Hypoxic preconditioning of stem cells has also shown promising results. It has been found to enhance graft survival after transplantation in a hemorrhagic stroke mouse model [163]. Results also revealed an increase in stem cell migratory and homing ability [164,165,166]. Cell survival and function are also boosted after transplantation [164]. Hypoxia contributes to the low graft survival occurring post-stroke transplantation. Rampant oxidative stress is also a significant contributor to graft and endogenous cell death [167,168]. Exposing stem cells to oxidative stress before transplantation has the potential to expand the survival time by allowing for genetic and phenotypic acclimation in an oxidative stress environment.

HBOT can also potentially increase stem cell secretome. In vitro experiments demonstrate that HBOT affects the secretion profile of stem cells. This involves proteins implicated in the oxidative stress response and proteins involved in neuroprotective pathways [160]. After HBOT, there is increased cellular nitric oxide which allows for the upregulation of growth factors, including VEGF and TFGb1 [160]. MSC cultures also demonstrated HBOT having the ability to enhance the expression of placental growth factor (P1GF). This was also correlated with increased MSC tubule formation and increased migratory ability [169]. HBOT has also revealed its ability to inhibit the differentiation of stem cells in culture [162]. Depending on the form of transplantation administered, the ability to promote stem cells may become a potential benefit of HBOT priming.

Research supports the evidence that HBOT-preconditioning on the healthy brain provides neuroprotective capacity. Stem cell preconditioning could also be used as a viable cell therapy strategy post-stroke. This could also be a viable and potent dual therapy technique administered to patients with a high risk for stroke. This strategy uses both a neuroprotective and neurorestorative approach.

The capability of HBOT to extend graft survival through oxidative stress conditioning, inhibit premature differentiation, augment migratory capacity, enhance injury homing, promote anti-inflammatory mediation, and upregulate trophic factors in the secretome demonstrate the potential of HBOT. HBOT is a viable therapeutic strategy that can work alone or combined with other preconditioning strategies to enhance the therapeutic efficacy of transplanted stem cells in the stroke brain.

### 6.4. Recent Literature on HBOT and Stroke

Recent literature has expanded knowledge of HBOT and its underlying mechanisms, all of which further elucidate its potential as a therapy for stroke, stroke-related symptoms, and other diseases. As an efficient and feasible treatment, HBOT has elicited neuroprotective effects before the stroke, displayed regenerative effects during the acute phase of stroke, and even alleviated symptoms during the chronic phase of the stroke. Furthermore, many diseases that mimic stroke pathology have found HBOT similarly effective.

#### 6.4.1. Preconditioning

Pretreatment with HBOT has been a focal point of research over recent years. An in vitro study examined and found that HBOT preconditioning of primary rat neuronal cells (PRNCs) mitigates cell death via mitochondrial transfer from astrocytes. PRNCs were subject to HBOT before exposure to tumor necrosis factor-alpha (TNF-alpha) or lipopolysaccharide (LPS) injury to induce stroke-like cell death. Upon examination, cell viability and mitochondrial transfer were both observed at augmented levels compared to the non-HBOT treated group. The ability to ameliorate both stroke-induced inflammation and cell death through preconditioning and mitochondrial transfer bolster this as a prophylactic therapy to prevent the devastating effects associated with stroke [178]. Another investigation elucidated the effects of HBOT on a rat model of permanent MCAO. HBOT lowered infarct volume and improved neurological scores in injured rats. An autophagy marker, Beclin-1, was seen at decreased levels after treatment. Expression of fodrin1 ceased and necrosis marker, PI-positive cells, were seen at decreased levels. TUNEL-positive cells, an indicator of apoptosis, were observed at reduced levels, and caspase-3 was downregulated. Taken together, this data indicate that HBOT may ameliorate the detrimental effects associated with ischemia through mitigating autophagy activity, apoptosis, and necrosis [179]. Another murine model featuring intracerebral hemorrhage investigated HBOT’s ability to attenuate edema, inflammation, and microglia activation. Pre-conditioning with HBOT was conducted for five days before ICH induction. MMP9 and brain edema were both less in the HBOT group when compared to control. Neuronal cell death and neurological deficits were minimized in the HBOT preconditioned group. Notably, the expression of M1 markers was reduced, consequently inhibiting microglia polarization and inflammatory pathways. This was apparent when measuring the concentration of pro-inflammatory cytokines, TNF-alpha, and IL-1β, with an indication that levels were downregulated. Furthermore, phosphorylation of JNK and STAT1 were significantly decreased in the HBOT group [180]. Lastly, a preconditioning combination therapy between melatonin (Mel) and HBOT was found to provide more favorable effects in the protection of ischemic injury-induced cognitive dysfunction and compromised parenchymal integrity in rats. Brain infarct area was lower in rats treated with HBOT-Mel than in rats treated with either HBOT or Mel monotherapy. Apoptotic, autophagy, and inflammatory markers indicated that combination therapy was more effective. The additional benefits provided by HBOT-Mel therapy warrant further investigation in the prevention of detrimental outcomes as a result of ischemic injury [181].

#### 6.4.2. Post-Stroke Treatment

On top of providing robust neuroprotective effects before a stroke, HBOT has also indicated efficacy in treating stroke patients’ post-ischemia. A study elucidated the effects of HBOT on chronic stroke patients who each underwent 40–60 sessions of HBOT therapy. Notably, 86% of patients displayed clinically significant improvements in cognitive function. When comparing cortical and hemorrhagic stroke victims, cortical stroke patients displayed heightened improvements in information processing. Data also suggested that baseline cognitive function should be contemplated rather than stroke type and location when predicting the magnitude of clinical improvements. [182]. Furthermore, upper limb motor dysfunction is a common debilitation after suffering a stroke. HBOT therapy, in combination with upper limb exercise and mental imagery (EMI), has shown promising results in clinical trials in improving outcomes of chronic stroke patients. When HBOT-EMI patients were compared to EMI patients alone, there were no statistically significant differences. However, HBOT-EMI patients showed an upward trend of improved motor function in upper limbs compared to the EMI group. Although not many differences were observed, data indicate that HBOT is a safe and practical therapy for chronic stroke patients, and this combination therapy should be explored further in the future [183]. Other than physical dysfunction, mental illness may manifest after stroke. A link has been found between HBOT and post-stroke depression (PSD), a common symptom many stroke patients experience. This is a devastating consequence of stroke, especially in other countries where it usually goes untreated. A clinical trial revealed an increased response rate and decreased depression scores post-HBOT. Furthermore, HBOT, in conjunction with antidepressants, was significantly more effective than each respective monotherapy. HBOT is a safe and feasible treatment to treat PSD; however, further elucidation is imperative [184].

#### 6.4.3. Diseases Resembling Stroke Pathology and HBOT

HBOT has been explored in treating diseases with pathological links to stroke. For example, TBI often presents with neuronal apoptosis, resembling stroke pathology. HBOT was investigated on a mouse model of TBI. Induction of TBI on mice resulted in activation of caspase-3, decreased levels of pGSK3β/GSK3β, pAkt/Akt, and β-catenin, and increased the prevalence of apoptotic neurons prior to HBOT. HBOT administration during the acute stage of TBI decreased apoptosis, possibly through attenuating the Akt/GSK3β/β-catenin pathway. Further investigation is necessary to fully comprehend the capacity of mediating this pathway and its implications within controlling TBI-induced apoptosis [173]. Cerebral air embolism, a phenomenon that complicates many medical procedures and can provide life-threatening symptoms, may be attenuated via HBOT. Cerebral air embolism pathology resembles that of stroke, often presenting with acute cerebral ischemia-induced edema. A patient undergoing a right internal jugular catheter procedure soon presented with fixed gaze palsy and left-sided hemiparesis upon removal of the wire. Imaging supported intraparenchymal air and a bubble in the right internal jugular vein. Soon after the manifestation of these symptoms, the patient underwent HBOT. Highly significant neurological improvements were seen over the course of the next week, indicating functional recovery. HBOT therapy may elicit ameliorative effects on central air embolism complications, and further investigation is warranted [185]. Lastly, HBOT efficacy was investigated in patients with hypoxic-ischemic encephalopathy (HIE). The subset of patients that received HBOT within nine months after the injury displayed the most significant results. This group showed improvements in the disorder of consciousness, as well as a more favorable coma recovery scale-revised score. Overall, HBOT within nine months after HIE may facilitate a functional recovery [186].

#### 6.4.4. Optimizing Treatment

Recent research advances have provided insightful information pertaining to HBOT and conducting the most efficient therapeutic strategy. Patients contemplating HBOT therapy may undergo SPECT/CT imaging as a predictor of HBOT efficacy. A patient analysis supported a link between large penumbra size and significant benefit from HBOT. This size was decreased substantially during therapy, and further supported by improved clinical neurologic status and better quality of life. Conversely, patients with small penumbra size did not significantly improve from HBOT, and the change penumbra size was negligible. This data suggests that patients presenting with large penumbra size may display more significant improvements than others. This imaging-based method of prediction may allow the ability to efficiently select patients that could benefit more from HBOT therapy [187]. Moreover, HBOT for prolonged periods of time can cause oxygen toxicity. It is important to evaluate effective protocols to ensure that this phenomenon does not occur during therapy. A murine model exhibited that intermittent hyperbaric oxygen exposure (IE-HBO) is linked to protection against oxygen toxicity. Continuous exposure (CE) of HBO increased concentrations of Peroxiredoxin 6 (Prdx6) protein, an endogenous antioxidant, indicating a relationship between the two. However, the IE-HBO group displayed higher amounts of Prdx6 in the rat brains and lungs compared to the CE-HBO group. IE-HBO also enhances NSGPx and GSH activity while mitigating oxidant formation in the lungs and brain. Taken together, IE-HBO mediated Prdx6 expression can suppress oxidative damage in the brain and lungs and overall protect against oxygen toxicity [188]. These findings allow for a safer and more effective treatment plan when utilizing HBOT to treat stroke and other diseases.

In all, HBOT presents with a multitude of treatment possibilities ranging from preconditioning to treating chronic disease. Moving forward, research should continue to explore this prophylactic therapy to better understand the underlying mechanisms that make it so effective. Similarly, treatment timing, session amount, and treatment duration should all be elucidated to establish optimal conditions to incorporate the best results.

## 7. Future Directions and Conclusions

HBOT has the ability to preserve vulnerable neural tissue and improve outcomes in stroke models, as seen in pre-clinical studies. However, further research needs to be conducted to find the most effective regimen of HBOT. Randomized controlled trials need to be administered in order to test human effectiveness. Even though further research regarding HBOT needs to be studied, current research has advanced our understanding of the mechanisms of HBOT, specifically in the injured and healthy brain. This knowledge has paved the way for the development of HBOT preconditioning strategies. Although HBOT preconditioning is a viable and innovative strategy that contains benefits for certain patients, limitations are present involving the ability to deliver stroke therapy.

HBOT has the ability to be applied as a preconditioning mechanism for stem cell transplantation. Research indicates that oxidative preconditioning of stem cell grafts through HBOT may be a viable strategy to promote graft survival and optimize graft function during the post-ischemic environment. In order to explore this concept, further research will be necessary regarding the genetic, epigenetic, secretome, and functional influence that HBOT exerts on stem cell populations. This potential therapeutic strategy would offer a hybrid approach of combining preconditioning strategies for neuroprotection in the ischemic state of the brain.

## Figures and Tables

**Table 1:** Chronological Reports of the Mechanisms Regulating HBOT-Induced Neuroprotection.

Study	Discovery
Jadhav et al., 2009	In surgical brain injury (SBI) mice, HBOT preconditioning ameliorated neurological function and cerebral edema; these neuroprotective effects seemed to be regulated by COX-2 mechanisms, as HBOT attenuated SBI-induced elevation of hypoxia-inducible factor-1alpha and COX-2 activity [170].
Mu et al., 2013	In permanent MCAO animal models, daily HBOT conditioning at 48 h post-surgery diminished infarct volume and improved neurological function, which correlated with elevated CREB protein expression in the hippocampus and peri-infarct area, boosting cell multiplication. Regarding acute pMCAo models, HBOT increased cerebral PP1-γ expression, alleviating CREB phosphorylation and ubiquitination spurred by ischemia. Moreover, HBOT’s regenerative effects against ischemic stroke can be associated with CREB and PP1-γ mechanisms [37].
Lu et al., 2014	In transient MCAO rat models, HBOT spurred an increase in ERK1/2 signaling due to higher levels of ROS, leading to the attenuation of autophagy. When U0126, an inhibitor of the ERK1/2 pathway, was applied, infarct size and autophagy were ameliorated [171].
Xue et al., 2016	MCAO rats subjected to HBOT preconditioning exhibited diminished infarct size, improved neurological behavior, and upregulated Sirt1, Nrf2, HO-1, and SOD1 expression, as well as reduction of MDA. Blocking of Sirt1 or Nrf2 abolished HBOT-induced protective effects, as Nrf2, HO-1, and SOD1 were repressed. Moreover, the protective actions of Sirt1, spurred by HBOT, may consist of the Nrf2/antioxidant defense mechanism [172].
Guo et al., 2016	Following successive HBOT pre-treatment over five days, rats underwent hyperglycemic MCAO. Preconditioning with HBOT significantly ameliorated hemorrhagic transformation induced by the Nod-like receptor protein 3 signaling and reduced infarct size, altogether rehabilitating neurological performance. HBOT’s neuroprotective effects could be linked to the ROS/thioredoxin-interacting protein/Nod-like receptor protein 3 mechanism [126].
Yang et al., 2017	HBOT ameliorated neurological impairment in TBI rats via upregulation of VEGF, VEGFR2, Raf-1, MEK1/2, and ERK1/2, stimulating proliferation of neural stem cells (NSC) and homing of these cells to the lesion site. The examination of HBOT’s protective effects in vitro showed similar results, as HBO drastically amplified NSC proliferation and VEGF/ERK signaling [123].
Hu et al., 2017	In hyperglycemia MCAO rats, exposure to two atmospheres of HBO for an hour immediately after dextrose administration ameliorated depleted ATP and nitcotinamide adenine dinucleotide levels, which in turn elevated silent mating type information regulation 2 homolog 1, alleviating cerebral infarct and neurological dysfunction, along with repressing hemorrhagic transformation [14].
He et al., 2019	Mice models of acute TBI demonstrated escalated levels of apoptotic neurons and caspase-3 activity, along with attenuation of signaling pathways that regulate apoptosis in neurons (e.g., pAkt/Akt, pGSK3β/GSK3β, and β-catenin). By eliminating the TBI-induced alterations in these pathways, HBOT suppressed neuronal apoptosis [173].
Ying et al., 2019	BDNF/TrkB signaling has been shown to influence rehabilitation after SCI. In vivo, SCI rat models were exposed to HBOT, and both dendritic/synaptic deterioration and apoptosis were ameliorated, which could be linked to higher levels of BDNF and TrkB activity. When ANA-12, an inhibitor of the BDNF/TrkB pathway, was administered, HBOT’s neuroprotective effects were reversed, indicating that HBOT’s therapeutic benefits are mediated by BDNF/TrkB signaling [174].
Zhou et al., 2019	Following HBOT, Sprague-Dawley rats with spinal cord injury (SCI) displayed ameliorated motor function and attenuated secondary injuries, such as inflammation and glial scar production. By blocking AKT and NF-kB signaling, HBOT repressed molecules associated with inflammation (iNOS and COX-2) and glial scar generation (GFAP and NG2) [175].

**Table 2:** Milestone Studies on the Use of HBOT for Stem Cells.

Study	Discovery
Yang et al., 2008	Rats were subject to unilateral carotid artery ligation and then 2 h of hypoxia. HBO2 was then administered following the hypoxic-ischemic event. The HBOT was found to upregulate neural stem cell proliferation in neurogenic environments within the adult brain [143].
Li et al., 2008	A murine model subjected rats to common carotid artery ligation and hypoxia for 90 min. HBOT was administered 24 h prior to the hypoxic-ischemic injury. Results revealed that rats preconditioned with HBOT had an increased survival rate, and the infarct ratio was decreased. This indicates that HBOT can provide brain protection via the inhibition of neuronal apoptosis pathways [45].
Li et al., 2009	HBOT preconditioned rats where investigated to determine if apoptotic inhibition through a mitochondrial pathway was correlated with neuroprotection in the ischemic injury in the rat brain. Preconditioning was conducted four times, followed by brain evaluation. Results indicated that HBO-PC significantly reduced brain edema and decreased infarction volume and improved neurological recovery [117].
Rink et al., 2010	Transient MCAO rodents outline the therapeutic potential of normobaric and hyperbaric oxygen treatments during ischemia and after ischemia. HBOT-treated rodents revealed inhibited leukocyte accumulation in the ischemic area due to a reduction in levels of inflammatory chemokines [50].
Cechin et al., 2014	This study allowed pancreatic progenitor cells to mature in a perfluorocarbon-based culture device that could adjust the levels of pO2. Enhanced O2 exposure in vitro led to maturation and differentiation of human embryonic stem cell-derived pancreatic progenitor cells [176].
Hadanny et al., 2015	Patients with cardiac arrest-induced chronic cognitive impairments where treated with sessions of HBOT and analyzed. After administering HBOT five days per week to chronic stroke patients, patients had significant improvements in memory and attention testing [22].
Dai et al., 2015	A rabbit model seeded human adipose-derived stem cells on a gelatin/polycaprolactone scaffold to determine the functional and histochemical improvement of tissue-engineered cartilage after HBOT. The human adipose-derived stem cells were found to have improved extracellular matrix-secreting abilities after transplantation into a rabbit cartilage defect model when primed with HBOT [177].
Yang et al., 2017	This study investigated the mechanism of HBOT that promote NSC proliferation and recovery following TBI. The study used 24 rats split into a sham group, a TBI group, and an HBO treated TBI group to determine the neurological differences. Neurological function was evaluated and monitored throughout the week. HBOT was found to promote neural stem cell migration to areas of injury within the brain in rat models of TBI that were preconditioned with HBO [123].
